# Supplementary material for: Inducible Enrichment of Osa-miR1432 Confers Rice Bacterial Blight Resistance through Suppressing OsCaML2
Source: Int J Mol Sci. 2021 Oct 21;22(21):11367. doi: 10.3390/ijms222111367 (PMC8583624; doi:10.3390/ijms222111367)
Supplement: Supplementary file 1 [file ijms-22-11367-s001.zip › ijms-1374736-supplementary.pdf]

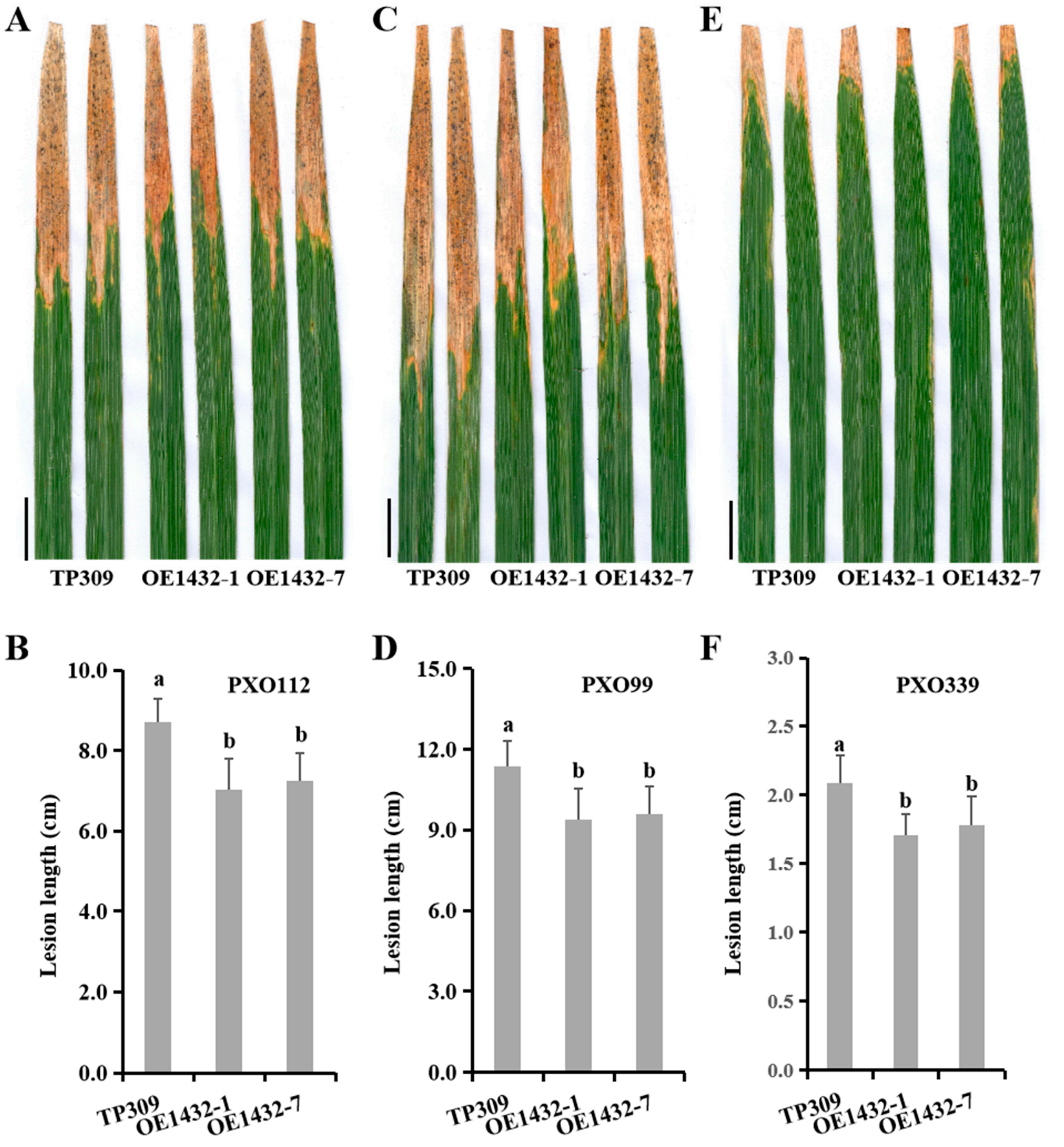

Figure S1. Disease phenotypes of osa-miR1432 overexpression transgenic lines caused by *Xoo* strain. (**A**) Lesion areas on OE1432 lines (OE1432-1 and OE1432-7) and the TP309 inoculated with bacterial blight PXO112. (**B**) Lesion areas on OE1432 lines (OE1432-1 and OE1432-7) and the TP309 inoculated with bacterial blight PXO99. (**C**) Lesion areas on OE1432 lines (OE1432-1 and OE1432-7) and the TP309 inoculated with bacterial blight PXO339. Scale bar, 2 cm. (**D**) Lesion lengths on OE1432 plants (n = 20) after 20 days of inoculated with bacterial blight PXO112. (**E**) Lesion lengths on OE1432 plants (n = 20) after 20 days of inoculated with bacterial blight PXO99. (**F**) Lesion lengths on OE1432 plants (n = 20) after 20 days of inoculated with bacterial blight PXO339.

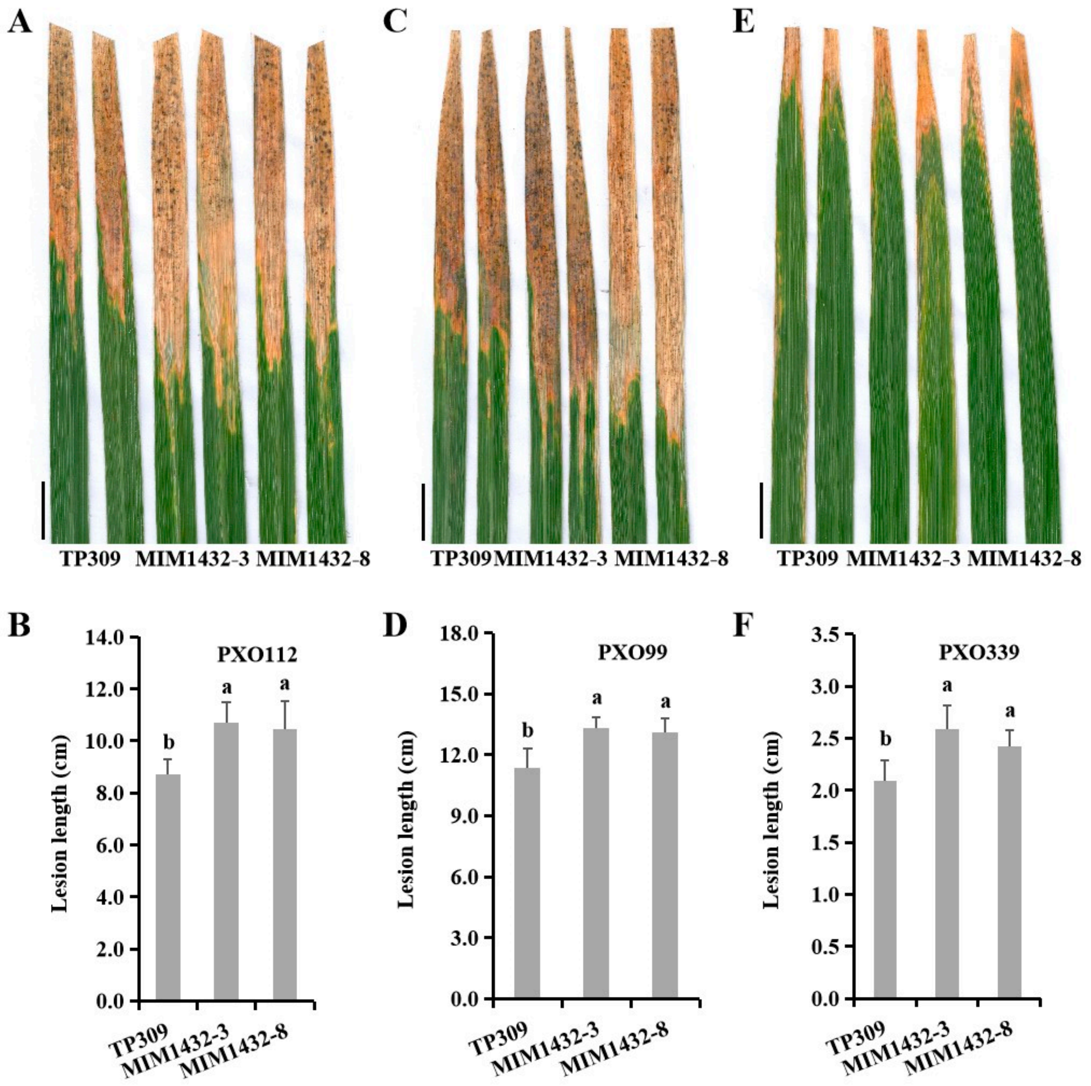

Figure S2. Disease phenotypes of MIM1432 overexpression transgenic lines caused by *Xoo* strain. (**A**) Lesion areas on MIM1432 lines (MIM1432-3 and MIM1432-8) and the TP309 inoculated with bacterial blight PXO112. (**B**) Lesion areas on MIM1432 lines (MIM1432-3 and MIM1432-8) and the TP309 inoculated with bacterial blight PXO99. (**C**) Lesion areas on MIM1432 lines (MIM1432-3 and MIM1432-8) and the TP309 inoculated with bacterial blight PXO339. Scale bar, 2 cm. (**D**) Lesion lengths on MIM1432 plants (n = 20) after 20 days of inoculated with bacterial blight PXO112. (**E**) Lesion lengths on MIM1432 plants (n = 20) after 20 days of inoculated with bacterial blight PXO99. (**F**) Lesion lengths on MIM1432 plants (n = 20) after 20 days of inoculated with bacterial blight PXO339.

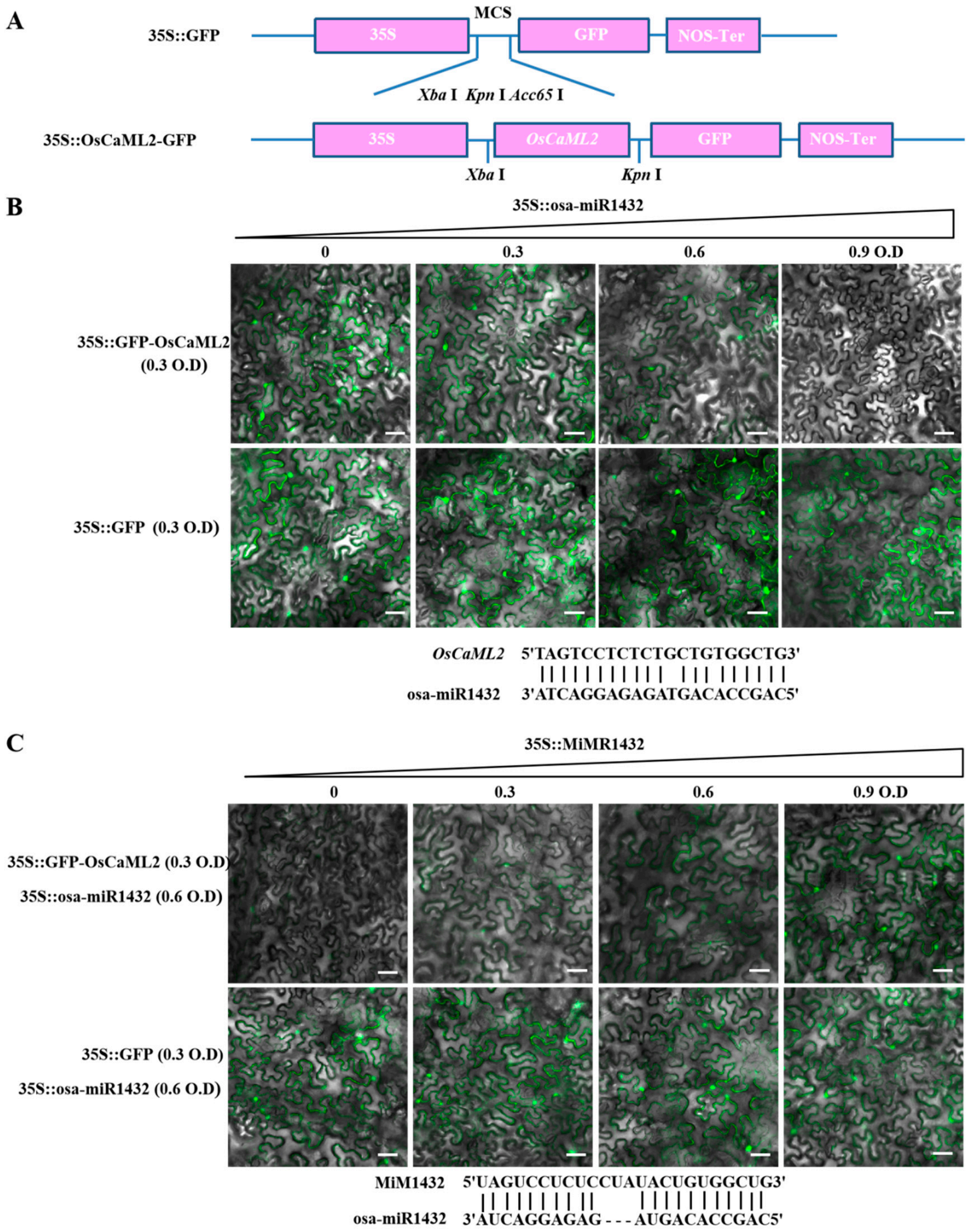

Figure S3. Targeting verification of osa-miR1432 and OsCaML2 using tobacco expression system. (**A**) 35S::OsCaML2-GFP vector construction. (**B**) The indicated 35S::OsCaML2 and GFP-based reporter constructs were expressed alone or co-expressed transiently with osa-miR1432 (**C**) or/and MIM1432 in Nicotiana benthamiana leaves using Agrobacterium-mediated infiltration at the indicated OD concentration. Scale bar, 50 μm.

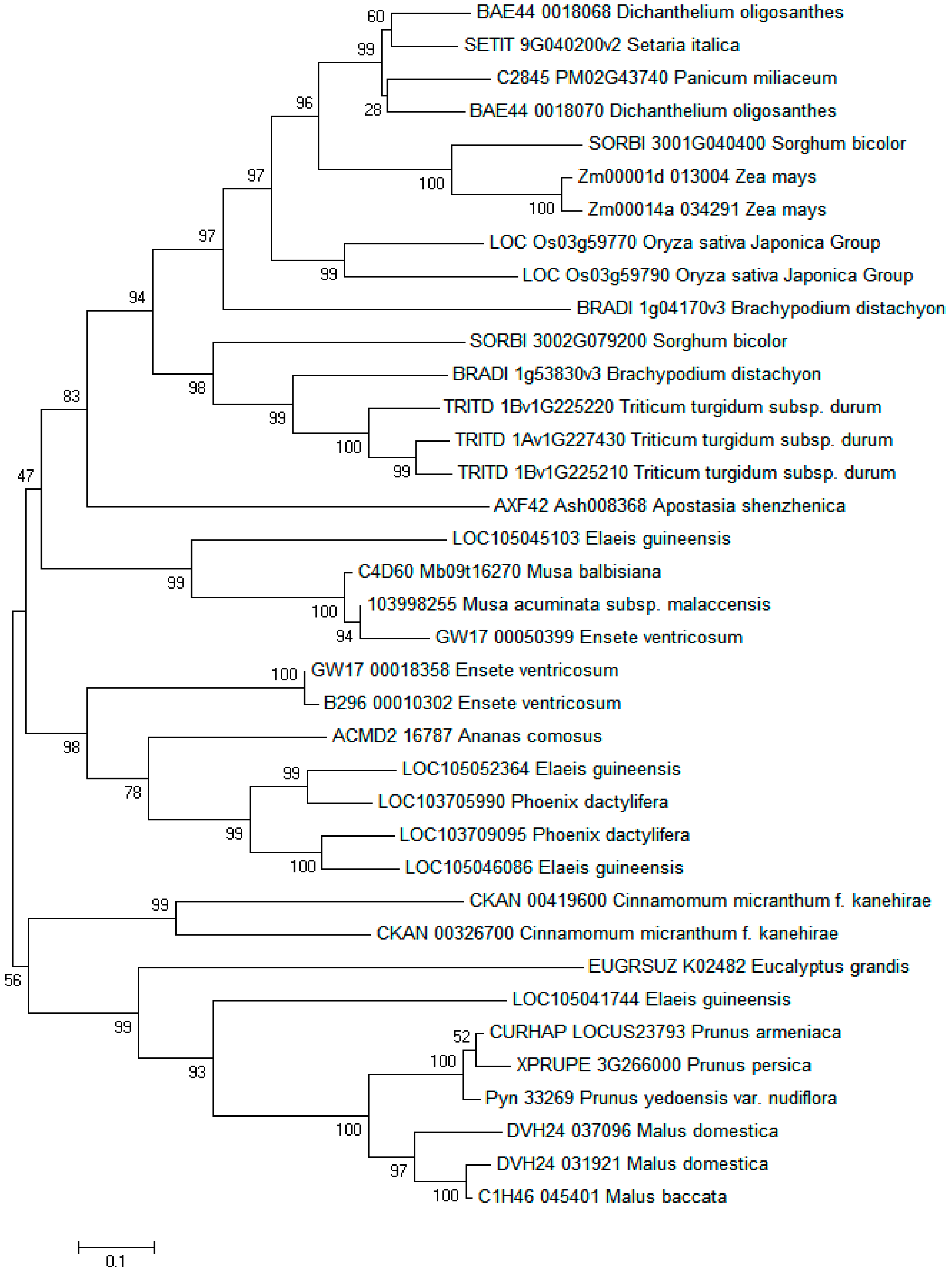

Figure S4. Phylogenetic analyses of putative homologs of OsCaML2. Phylogenetic analysis of putative OsCaML2 homologs using MEGA with neighbor method, bootstrap analysis was performed with 1000 replicates and excluding positions with gaps. Numbers in branches indicate bootstrap values (percent).

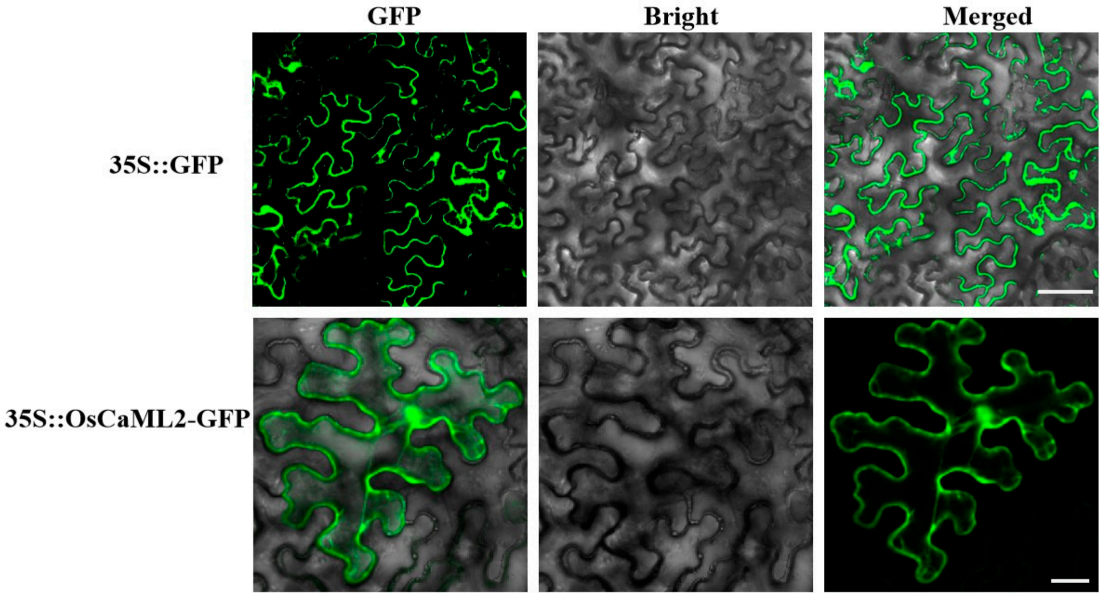

Figure S5. Subcellular localization of OsCaML2. Bar, 40 μm.

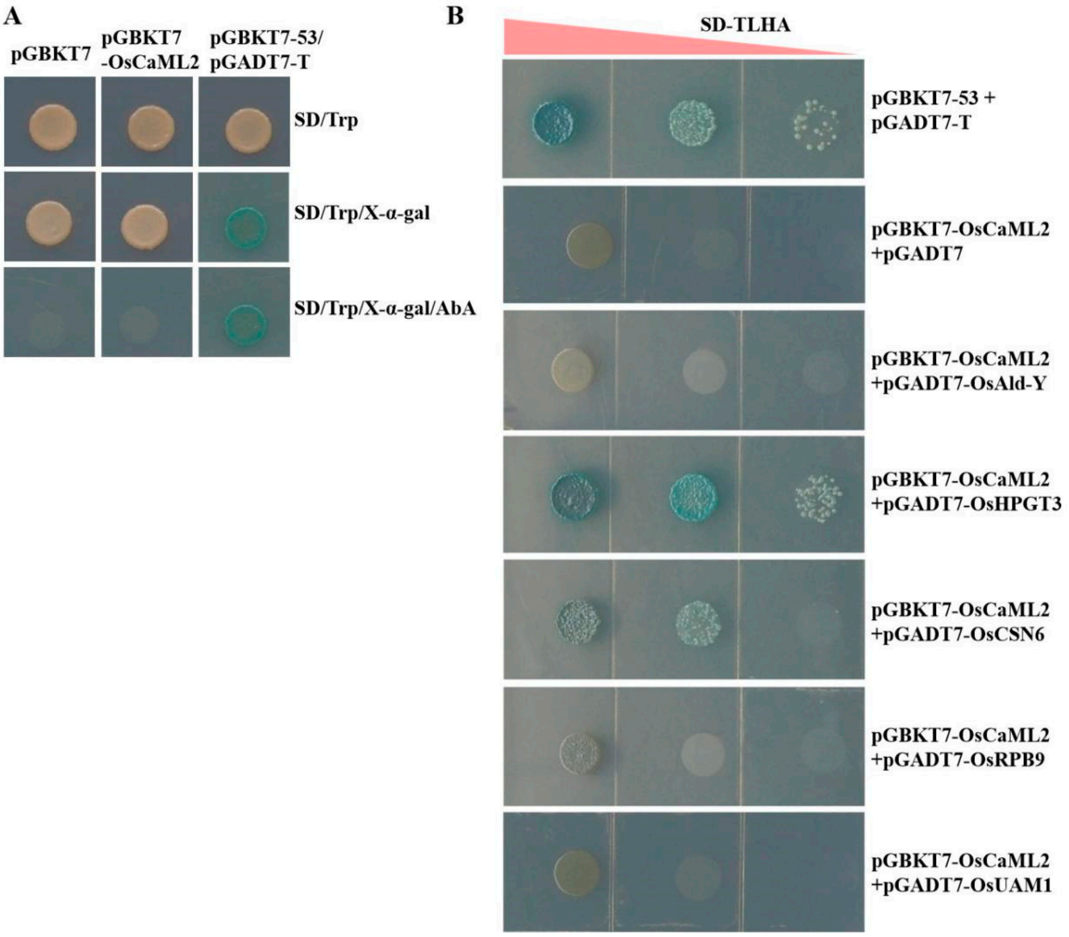

Figure S6. Screening of the interacting proteins of OsCaML2. **(A)** The self-activation and toxicity analysis of OsCaML2 protein. pGBKT7: negative control, pGBKT7-53/pGADT7-T: positive control. **(B)** Screening of the interacting proteins of OsCaML2 by Y2H. pGBKT7-OsCaML2 + pGADT7: negative control, pGBKT7-53 + pGADT7-T: positive control. Red arrows indicate dilution multiples of 1, 10 and 100, respectively.

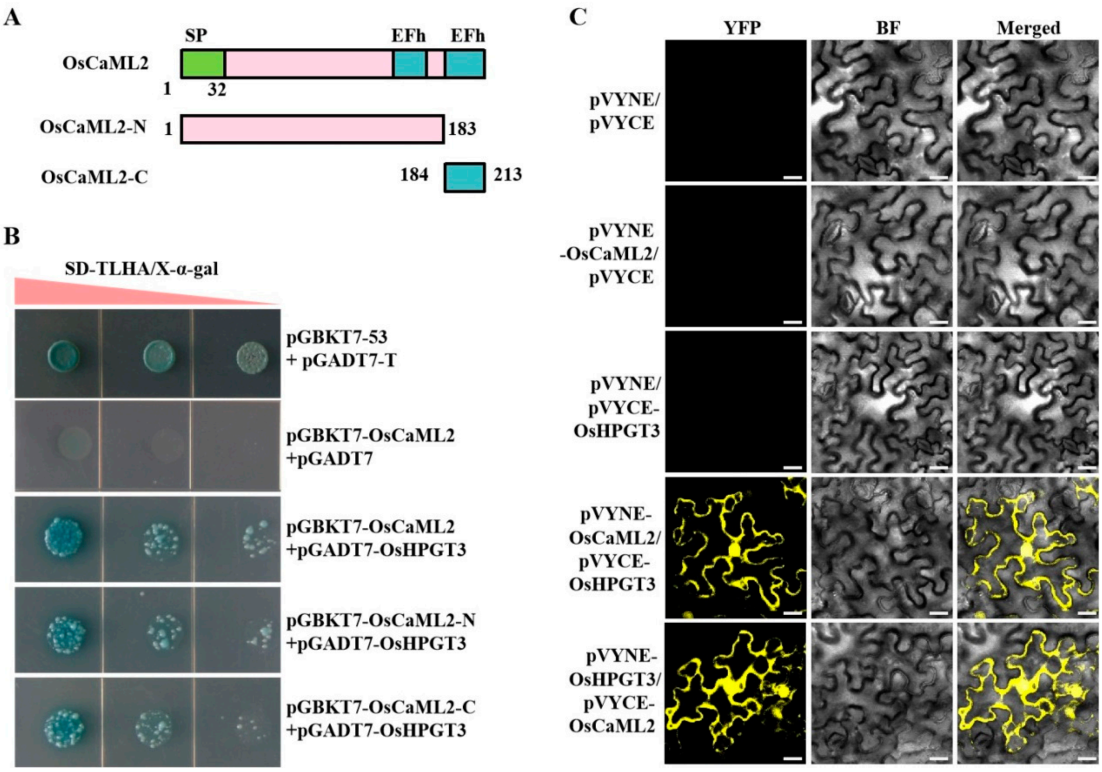

Figure S7. Y2H and BiFC assay of interaction between OsCaML2 and OsHPGT3. **(A)** Schematic diagram of OsCaML2 full length and truncated sequence. **(B)** Analysis of interaction between full length, truncated OsCaML2 and OsHPGT3 in yeast cell. **(C)** Validation of interaction between OsCaML2 and OsHPGT3 in tobacco leaf cell. Bar, 40 μm.

Table S1. Primers used in this study.

Name	Primer sequence (5′-3′)	Purpose
Osa-miR1432-F	CGAAGCTTGTGGTTGACTCCGCAACCTA	for cloning
Osa-miR1432-R	CCGGTACCGAGATGAGGGAGGATGTGCG	for cloning
IPS1-F	GGGGTACCCCTGGCCATCCCCTAGCTAGGT	for cloning
IPS1-R	GGGGTACCCCTGGCCATCCCCTAGCTAGGT	for cloning
MIM1432-F	GTCGGTGTCATCTACTCTCCTGATAGCTTCGGTTCCCCT	for cloning
MIM1432-R	GTCGGTGTCATCTACTCTCCTGATAGCTTCGGTTCCCCT	for cloning
OsCaML2-F	GAACACAACACAAACACATCGC	for cloning
OsCaML2-R	CCATCTGGAACGCTGAAATAGT	for cloning
pCAMBIA1300-osa-miR1432-F	GGTGTTACTTAGGCTTGTGGTTGACTCCGCAACCTA	vector construction for transgene
pCAMBIA1300-osa-miR1432-R	TGTAGTCCATGGTACCGAGATGAGGGAGGATGTGCG	vector construction for transgene

pCAMBIA1300-OsCaML2-F	GGTGTTACTTAGGCTTATGTCGCAGTTTGTGGCGACGTTC	vector construction for transgene
pCAMBIA1300-OsCaML2-R	TGTAGTCCATGGTACCCTAAACCGCGTTCTCCATCATGGCTCT	vector construction for transgene
OsPR1a-qRT-F	TGCTATGCTACGTGTTTATG	qPCR
OsPR1a-qRT-R	AAATACGGCTGACAGTACAG	qPCR
OsPR1b-qRT-F	GGCAACTTCGTCGGACAGA	qPCR
OsPR1b-qRT-R	CCGTGGACCTGTTTACATTTTCA	qPCR
OsPBZ1-qRT-F	CGCAAGTCATGTCCTAAAGTCG	qPCR
OsPBZ1-qRT-R	ATGCCATAGTAGCCATCCACG	qPCR
OsACTIN1-qRT-F	CCTGACGGAGCGTGGTTAC	qPCR
OsACTIN1-qRT-F	CCAGGGCGATGTAGGAAAGC	qPCR
Osa-mi1432-qRT-F	TCGCTATCAGGAGAGATGAC	qPCR
Universal-qRT-R	GTGCAGGGTCCGAGGT	qPCR

U6-qRT-F	GGGGACATCCGATAAAATTGG	qPCR
U6-qRT-R	ACCATTTCTCGATTTGTGCGT	qPCR
OsCaML2-qRT-F	GACGTTCGAGTACTGCAGCC	qPCR
OsCaML2-qRT-R	GCTCTGGACATGACGAGGAT	qPCR
OsCAS-qRT-F	AGGTCACTGAAGGATGTGGG	qPCR
OsCAS-qRT-R	TCAACCCCTTCTTTTCAGCA	qPCR
OsZIP6-qRT-F	GCAGGCAGGTTTTGGGATTG	qPCR
OsZIP6-qRT-R	GAAGAAAGTGAGCCAAGGAGC	qPCR
pGBKT7-OsCaML2-F	GCCATGGAGGCCGAATTCATGTCGCAGTTTGTGGCGACG	Y2H
pGBKT7-OsCaML2-R	GTTATGCGGCCGCTGCAGCTAAACCGCGTTCTCCATCAT	Y2H
pGBKT7-OsCaML2-C-R	GTTATGCGGCCGCTGCAG CTACAGCCTCCGCATCACGTT	Y2H
pGBKT7-OsCaML2-N-F	GCCATGGAGGCCGAATTC ATGGGCATTGAGGAGGGCGCG	Y2H

pGADT7-OsCaML2-F	GGAGGCCAGTGAATTCATGTCGCAGTTTGTGGCGACGTTC	Y2H
pGADT7-OsCaML2-R	TCATCTGCAGCTCGAGCTAAACCGCGTTCTCCATCATGGC	Y2H
pGADT7-OsCSN6-F	GGAGGCCAGTGAATTCATGTCGGCGCCATCCGACCCCGCC	Y2H
pGADT7-OsCSN6-R	TCATCTGCAGCTCGAGCTACATGAAAGCACCTCGGCCTCC	Y2H
pGADT7-OsHPGT3-F	GGAGGCCAGTGAATTCATGGAGACGCTGGCGAGCGCCATG	Y2H
pGADT7-OsHPGT3-R	TCATCTGCAGCTCGAGTCATGCGTGGGAACATACTTTCTC	Y2H
pGADT7-OsUAM1-F	GGAGGCCAGTGAATTCATGGCGGGGACGGTGACGGTGCCG	Y2H
pGADT7-OsUAM1-R	TCATCTGCAGCTCGAGCTACTTGGCCTTGCCGTTCTCGAC	Y2H
pGADT7-OsRPB9-F	GGAGGCCAGTGAATTCATGAGCACCATGAAGTTTTGCCGC	Y2H
pGADT7-OsRPB9-R	TCATCTGCAGCTCGAGTCATTCCCTCCATCGGTGGGCGCA	Y2H
pGADT7-OsAld-Y-F	GGAGGCCAGTGAATTCATGTCTGCCTTTGTGGGAAAATAC	Y2H
pGADT7-OsAld-Y-R	TCATCTGCAGCTCGAGCTAGTAGGTGTAGCCCTTGACGTA	Y2H

pVYNE/CE-OsCaML2-F	CCCAGGCCTACTAGTGATGGGCATTGAGGAGGGCGCG	BiFC
pVYNE/CE-OsCaML2-R	CTCCTACCCGGGAGCGCTAAACCGCGTTCTCCATCATGGC	BiFC
pVYNE/CE-OsHPGT3-F	CCCAGGCCTACTAGTGATGGAGACGCTGGCGAGCGCCATG	BiFC
pVYNE/CE-OsHPGT3-R	CTCCTACCCGGGAGCGTGCGTGGGAACATACTTTCTCTTG	BiFC
35S::*OsCaML2*-GFP-F	CACGGGGGACTCTAGAGATGTCGCAGTTTGTGGCGACG	YFP-based reporter assay
35S::*OsCaML2*-GFP-R	CCTTACCCATGGTACCAACCGCGTTCTCCATCATGGC	YFP-based reporter assay
35S::MIM1432-F	CACGGGGGACTCTAGAGACCCTCTCTTAACTTGGCAAA	YFP-based reporter assay
35S::MIM1432-R	ATCGGGGAAATTCGAGCTCGAGGAATTCACTATAAAGA	YFP-based reporter assay
35S::osa-miR1432-F	CACGGGGGACTCTAGAGGTGGTTGACTCCGCAACCTAT	YFP-based reporter assay
35S::osa-miR1432-R	ATCGGGGAAATTCGAGCTCGAGATGAGGGAGGATGTGCG	YFP-based reporter assay

Table S2. Potential targets of osa-miR1432 predicted by psRNATarget.

Target ID	Expectation	UPE	Description	Inhibition

LOC_Os03g59790	0.5	23.499	calcium-binding protein	Cleavage
LOC_Os03g59770	0.5	20.601	Calcium-binding protein CML21-like	Cleavage
LOC_Os04g08350	3	18.823	Cysteine synthase, chloroplast/chromoplast precursor CAS2	Translation
LOC_Os04g35590	3	19.577	Thioesterase family protein	Cleavage
LOC_Os05g07210	3	11.004	ZIP zinc/iron transport family protein	Cleavage
LOC_Os08g36910	3	11.966	alpha-amylase precursor	Cleavage
LOC_Os10g30540	3	19.001	Lectin-like receptor kinase	Cleavage
LOC_Os06g40940	3	11.989	Glycine dehydrogenase	Cleavage
